# Occupational disease predictors in the nickel pyrometallurgical production: a prospective cohort observation

**DOI:** 10.1186/s12995-022-00362-2

**Published:** 2022-11-05

**Authors:** Sergei Syurin, Denis Vinnikov

**Affiliations:** 1 Northwest Public Health Research Center, 4 2-Sovetskaya street, 191036 Saint-Petersburg, Russian Federation; 2 al-Farabi Kazakh National University, 71 al-Farabi avenue, 050040 Almaty, Kazakhstan; 3 Peoples’ Friendship University of Russia (RUDN University), 6 Miklukho-Maklaya street, 117198 Moscow, Russian Federation

**Keywords:** Nickel production, Pyrometallurgy, Occupational diseases, Compensation claims, Cox regression

## Abstract

**Background:**

Pyrometallurgical nickel production exposes workers to a wide range of occupational risk factors, including nickel aerosol, occupational noise and heat, but occupational (compensation) claims do not get enough attention in the literature. We, therefore, aimed to identify and analyze new occupational disease predictors in order to tailor prevention measures in the nickel pyrometallurgical production workers.

**Methods:**

In a prospective observational study, a cohort of workers grouped in 16 occupations (N = 1424, 88% males, median age 39 (interquartile range (IQR) 31–47 years)), was fixed in 2007 at a large nickel production plant in the Russian High North. We then followed the cohort until 2021 and analyzed the association of selected predictors, including exposure to nickel and occupational group, with the risk of an occupational (compensation) claim in a Cox regression analysis.

**Results:**

With 18,843 person-years of observation, occupational disease claims were confirmed in 129 workers (9% of the initial cohort, N = 108 men (84%)). Top three diagnoses were chronic bronchitis (3.81 cases/1000 workers/year), sensorineural deafness (2.36 cases/1000 workers /year) and musculoskeletal disorders (1.90 cases/1000 workers/year). Smoking was significantly associated with each diagnosis (adjusted hazard ratio (HR) ranged from 2.56 (95% confidence interval (CI) 1.17–5.57) for bronchitis to 6.69 (95% CI 1.46–30.64) for chronic obstructive pulmonary disease (COPD)). High nickel exposure was associated with occupational bronchitis and occupational asthma, whereas associations of occupational groups were also identified for COPD, asthma and musculoskeletal disorders.

**Conclusion:**

Smoking, high exposure to nickel and specific exposure in the occupational groups increase the risk of occupational disease claims and should be prioritized directions for targeted intervention.

## Background

Nickel production is usually a multi-stage process of ore mining, pyrometallurgical processing and electrolysis, when workers can be exposed to a varying combination of metal itself with its compounds in the form of dust or aerosol, with inhalational or dermal routes of exposure. Such exposure may vary dramatically from one workplace to another, whereas health effects of exposure are usually guided by the water-solubility of nickel salts and compounds.

Nickel is known for toxic, carcinogenic and allergic effects, and exposure to a range of nickel compounds and metallic nickel may occur in various industries [1, 2]. Allergic reactions to nickel have been reported even in those never exposed in the workplace [3, 4], but most chronic effects of long-term exposure, including toxic effects and malignancies [5], have been described in the occupationally exposed humans. High lung and nasal cancer risk is usually related to less soluble oxidic and especially sulfidic nickel species in refinery dust [6]. Molecular mechanisms of nickel-induced toxicity have yet to be fully understood, mitochondrial dysfunctions and oxidative stress have been demonstrated to have a primary role in the nickel toxicity [7]. Insoluble nickel salts are generally considered more toxic compared to water-soluble compounds [6, 8].

Because inhalational route is predominant in nickel production, respiratory disease, including chronic bronchitis and chronic obstructive pulmonary disease (COPD) take the lead in the occupational morbidity profile in nickel mining and pre-electrolytical processing [9]. However, scientific data with analysis describing exposure-response association in this industry are still limited and sometimes inconsistent. The presentation of confirmed occupational disease and compensation claims in those exposed to nickel could be one of the ways to describe and understand the burden of nickel in occupationally exposed workers. We have earlier reported occupational disease compensation claims in nickel electrolysis workers from one of the world leading nickel final product producers, where we have identified a greater risk in the final product cleaners [10].

Occupational disease predictors and risk factors in the cohorts of workers employed for processes prior to electrolysis, including ore processing and pyrometallurgic stages, have never been analyzed. Given that exposures at this stage of nickel production may be specific and are a combination of industrial aerosol, noise and heat, and that there exists a specific system of grading work environment in the Russian Federation and the former Soviet Union countries [11], we wanted to differentially assess occupations in the nickel pyrometallurgical production with regard to occupational claims with the elapsing time of employment. Of note, work conditions assessment in the Russian Federation is performed by the governmentally certified organizations and implies stratification of workplaces into four hazard grades, depending on the occupational exposure limit (OEL) exceedance. Therefore, the aim of this analysis was to identify and analyze new occupational disease predictors in order to tailor prevention measures in the nickel pyrometallurgical production workers.

## Methods

### Study design, cohort construction and its occupational profile

This was a prospective observation of all enlisted employees of a nickel pyrometallurgical production site of one of the leading Russian nickel producers, situated in the Russian High North. The company reported production of 166,265 tons of nickel (approximately 10% of the total world production) in 2019. The company is one of the largest nickel producers in the world and sustains full nickel production cycle at various sites, including ore mining, ore dressing, pyrometallurgical processing and nickel refining by electrolysis and carbonyl methods. We earlier analyzed and described occupational claims of workers at another nickel production site of the same company, employed in the nickel electrolytic production [10]. Unlike electrolysis, usually considered the final stage of ready product production, pyrometallurgical ore processing stands midway between mining and final product. Because occupational exposures, physical site location and technologies were basically different throughout the production cycle, cohorts of electrolysis workers presented earlier, and the current pyrometallurgical processing cohort were analyzed separately.

This study received approval from the Committee of Bioethics of the Northwest Public Health Research Center and was conducted in accordance with the relevant guidelines and regulations. At the annual screening described below (mandatory for employees under Russian law), all employees signed a written informed consent to participate and to have the obtained data used for research purposes. There were no workers who refused to participate.

Once constructed by the end of annual screening in 2007, the study cohort was fixed and followed for subsequent years till 2021. All enlisted at the pyrometallurgical site employees were referred to the annual screening by the official Order of the human resources department. Therefore, annual screening of 2007 was considered as time 0 for this observational study. We tracked all new cases of occupational disease (compensation) claims leading to contract discontinuation, as well as all other reasons of drop-out annually using workers’ personal IDs. All cases of occupational disease (compensation) claims were obtained from the central database of the Kola branch of the Northwest Public Health Research Center, which also acted as the Provincial Center of Occupational Disease, legally in charge of occupational claims verification.

In total, workers from 54 workplaces of pyrometallurgical processing site were included and grouped into 16 occupational groups. As of the legislation in occupational health in 2007, work conditions assessment in the industry was part of workplace attestation performed by the governmentally certified organizations, such as Kola branch of the Northwest Public Health Research Center once every five years. In addition, governmentally certified in-house analytical laboratory was in charge of routine work conditions assessment four times a year. Comprehensive exposure assessment as part of such work conditions assessment implied classification of hazard grade in each workplace depending on the fold-range of OEL exceedance [11]. Of note, out of five occupational factors (Table 1), fixed OEL existed only for four occupational factors, whereas OELs for chemicals varied depending on a specific substance. The current classification assumed four hazard grades: (1) optimal (below OEL); (2) acceptable (below or equal to OEL); (3) hazardous, with four subgrades (exceeding OEL in all sub-grades with the corresponding fold-range); (4) dangerous (above OEL and with the fold-range higher than in 3.4) (Table 1).

**Table 1:** Hazard grading classification with regard to OEL exceedance

Hazard	Hazard grade (3)				Dangerous grade (4)
	3.1	3.2	3.3	3.4	
Chemicals (OELs vary between substances)	1.1-3.0 OELs	3.1–10.0 OELs	10.1–15.0 OELs	15.1–20.0 OELs	> 20.0 OELs
Low fibrogenic aerosols (OEL > 2 mg/m^3^)	1.1-3.0 OELs	3.1-6.0 OELs	6.1–10.0 OELs	> 10.0 OELs	none
Whole body vibration (OEL 0.1 m/s^2^)	2 OELs	4 OELs	6 OELs	8 OELs	> 8 OELs
Hand-arm vibration (OEL 2 m/s^2^)	1.4 OELs	2 OELs	2.8 OELs	4 OELs	> 4 OELs
Noise (OEL 80 dBA)	5 dBA	15 dBA	25 dBA	35 dBA	> 35 dBA

*Note: OEL – occupational exposure limit*

Compared to hazard grading classification with regard to OEL exceedance, the methodology to define hazard grades with regard to labor intensity and workplace microclimate was more complex and included dynamic and static workload, motions stereotype, lifting and moving weight and work posture. Hazard grading as of the labor intensity was defined as a function of intellectual, sensor and emotional load, as well as their monotony. Manual exertion of nickel production workers, as reported by the company, was usually within the existing recommendations, differential for males and females, which considered 14 measured indicators. These indicators included single weight to lift (below 30 kg); repeated weight to lift (below 15 kg); the overall hourly weight moved from the working surface (below 870 kg); the overall hourly weight moved from the floor (below 435 kg); the overall count of repeated local movements during the shift for palm fingers (below 40,000); the overall count of repeated regional movements during the shift for arm muscles (below 20,000).

In addition, workplace microclimate assessment was based on the temperature, humidity, air flow and work intensity with four corresponding hazard grades [11]. Such approach of workplace assessment allowed to mold fifteen occupational groups, including smelters (group 1), metalworkers (group 2), electricians (group 3), crane operators (group 4), burners (group 5), welders (group 6), metal cleaners (group 7), foremen (group 8), riggers (group 9), crusher operators (group 10), batchers (group 11), gas purification operators (group 12), converter operators (group 13), flotation operators (group 14), mil operators (group 15), and in addition a versatile group of “others” (group 16). The greatest OEL exceedance was noted for chemical substances and low fibrogenic aerosols, which made the greatest contribution to the overall grade along with labor severity. In general, smelters, burners, crusher and converter operators were graded as most hazardous workplaces with 3.4-4 hazard grades, as shown in Table 2.

**Table 2:** Hazard grades of included occupations for each hazard and overall

Workplace (occupation)	Chemicals	Low fibrogenic aerosols	Whole body vibration	Hand-armvibration	Noise	Labor severity	Labor intensity	Cooling microclimate	Overall
Smelter	3.4	3.3–3.4	2-3.1	3.1–3.2	3.2	3.2	2	3.3	3.4-4
Metalworker	3.3	3.3	2	2	3.1	3.2	2	3.1	3.3–3.4
Electrician	3.3	3.2–3.3	2	2	3.1	3.1	2	3.1	3.3
Crane operator	3.3	3.3	2	2	3.1	3.1	3.1	2	3.3–3.4
Burner	3.4	3.4	2-3.2	2	3.2	3.1	2	3.1	3.4-4
Welder	3.2	3.1	2	2	3.2	3.1	2	3.1	3.3
Metal cleaner	3.2	3.2	2	2	3.2	3.1	2	3.1	3.3
Foreman	3.2–3.3	3.1	2	2	3.1–3.2	2	3.1	3.1	3.2–3.3
Rigger	3.2–3.3	3.1	2	2	3.2	3.1–3.2	3.1	3.1	3.3
Crusher operator	3.3	3.3-4	2	2	3.2–3.3	3.1	2	3.1	3.3-4
Batcher	3.3	3.2–3.3	2	2	3.2	3.1	2	3.1	3.3
Gas purification operator	3.3	3.2	2	2	3.2	3.1	2	3.1	3.3–3.4
Converter operator	3.3-4	3.3	2	2-3.1	3.1	3.2	2	3.3	3.3-4
Flotation operator	3.3	3.1	2	2	3.2	3.1	2	3.2	3.3
Mill operator	3.2–3.3	3.3	2	2	3.2	3.1	2	3.1	3.3–3.4

### Annual screening

Annual screening was conducted in accordance with the Russian Federation Order of the Ministry of Health, which dictated panel composition, timing, legal assumptions, procedure to inform employer, and listed mandatory medical examinations and tests along with contraindications to work. Workers were referred to the annual screening to the Kola branch of the Northwest Public Health Research Center. Eight specialists of the panel, who screened health status, identified medical conditions and diseases and verified medical contraindications to work were reinforced by a pulmonologist, endocrinologist, gastroenterologist and urologist. Upon screening completion, an employee was expected to have a fitness certificate, whereas the medical profile was stored, and depersonalized data were transferred and analyzed afterwards. The panel should identify medical conditions and confirm diagnosis with routine (electrocardiography, spirometry, X-ray, audiometry) and specific ancillary examinations, including cold and vibration tests. Should an employee exhibit signs suspicious of a work-related disease, he or she is then referred to an advanced medical examination in the specialized facility. For workers employed in the nickel production industry, the Order mandates screening once a year; the screening protocol from 2007 did not change until the study completion. Diagnoses from the annual screening in 2007, at that time treated as general chronic conditions and not work-related yet, were analyzed as predictors for occupational disease claims in the current presentation along with other occupational and behavioral (smoking) variables.

### Air nickel concentrations

Nickel chemical compounds in the breathing zone may act as a major occupational chemical hazard in those employed for nickel pyrometallurgic processing and may include metallic nickel, nickel oxides and sulfides, nickel compounds mixtures called stein and fine stein, nickel concentrate and agglomerate, cleaning device dust, nickel compounds aerosol and nickel with chromium. Air sampling was routinely performed by a certified in-house chemical laboratory in selected workplaces at least once every three months. As of the current legislation, sampling can only be performed by the certified laboratories and only in compliance with the nationwide protocol. This protocol implies area sampling in locations with close proximity to emission sources and locations with expected high exposure, such as electrolysis baths, blast furnaces, etc. This sampling yields records of the mean, minimal and maximal concentrations, as well the percent of samples exceeding OEL. In addition to routine sampling of cobalt, copper, lead, arsenic anhydrides, formaldehyde, sulfur dioxide, carbon monoxide, ammonia, sulfuric acid and overall dust, nickel was quantified in samples using polarographic method and ПУ-1 device. The air in selected locations was aspirated using А-01 or АМ-5 pumps. As of the current protocol, nickel, nickel oxides and sulfates were quantified in the dust samples precipitated on АФА-ВП20 filters from 120 l (pump speed 10 l/min), or from 1000 l of air (pump speed 35 l/min) for water-soluble compounds. We extracted data on routine nickel sampling data from the company records and averaged four quarterly concentrations in a given year for each specific workplace analyzed in this study. As of the current legislation, OEL for water-insoluble nickel compounds was 0.05 mg/m^3^ and 0.005 mg/m^3^ for nickel hydroaerosol.

### Statistical analysis

The primary outcomes of interest were absolute number of cases of occupational disease claims overall and each year during the follow-up. These cases are also presented as relative measures of effect when divided by the total numbed of occupational disease claims and, alternatively, to the overall number of workers initially included in the cohort. All baseline demographic data as continuous variables, including age, years in service and air nickel concentrations were tested for normality using Shapiro-Wilk test. Binary variables, such as sex distribution, are expressed as N with the corresponding percent from the overall count. Because most continuous variables were non-normally distributed, we used nonparametric Mann-Whitney U-test to compare two groups and Kruskall-Wallis test for three or more groups. Binary variables between groups were tested using contingency tables and the corresponding χ^2^ test. If not shown otherwise, we reported medians with the associated interquartile range (IQR). Whenever data were normally distributed in the group, we reported means with standard deviations.

We first tested the difference in the major continuous and binary variables between sixteen occupational groups, as specified above, as of the annual screening in 2007, representing cross-sectional analysis. We then documented the new cases of occupational disease claims during the follow-up and reported the incidence rates as number of incident cases per 1000 workers per year for each specific occupational diagnosis. Air nickel concentrations, first available as continuous variables, were tested as a predictor for occupational disease claims (described below), but were recoded to a binary variable of high vs. low nickel exposure. To determine the cut-off level, we used receiver operating curves (ROC)-analysis, in which an air nickel cut-off level with the greatest (sensitivity + specificity) was obtained in addition to reported area under the curve (AUC) with its 95% confidence interval (CI) and the associated p-value. In the subsequent analyses, exposure to nickel was treated as a binary variable of high vs. low concentrations using the obtained cut-off value.

The secondary outcome of interest in the current analysis was the chance (probability) to obtain a confirmed occupational disease (compensation) claim, first overall, and then for a specific diagnosis, in crude, and then adjusted Cox regression models. Selected predictors were chosen from the annual screening in 2007, including occupational groups (one of fifteen groups, because group 16 was excluded from the later analyses), whereas the “time” variable in the regression model was the elapsed time since the start of employment (the overall work duration) to either fail, such as in case of an occupational disease claim, or censor, should this case not happen. These models reported hazard ratios (HR) with the corresponding 95% CI in the adjusted models as specified in each specific case or for a specific occupational diagnosis. Predictors for adjusted Cox regression models were chosen depending on the crude comparisons. Smoking in all presented models was included as a binary variable (yes/no); and the alternative analysis with pack-years did not alter the effect (data not presented). Among other predictors, nickel exposure and occupational groups were tested in the adjusted model to see whether their effects were independent of each other, despite some nickel exposure present in most groups. All tests were accomplished in NCSS 2021 (Utah, USA), and p-values below 0.05 were considered significant.

## Results

### Sociodemographic data

In 2007, when the study cohort was constructed, 1424 nickel production workers (1249 or 88% males) who completed annual or pre-employment screening, were included in the current analysis and followed until 2021 (Table 3). These 1424 workers made 93% of the overall listed staff of the nickel pyrometallurgic production department, and the remaining 7% who missed the annual screening, were not analyzed hereinafter. 88% of the cohort were males, only 15% were considered healthy with no diagnoses at the annual screening and almost 60% were daily cigarette smokers. Smelters, metalworkers and electricians were top three most prevalent occupations, making 42% of the staff altogether. Median age at inclusion was 39 (IQR 31–47) years with the median work duration 14 (IQR 8–22) years. As expected, age and work duration in the current position were highly correlated (Pearson r = 0.81 (95% CI 0.79–0.83)), because most workers would stay in their workplace within the company for the most of their career in the absence of other industrial employers nearby.

**Table 3:** Demographic and occupational profile of the studied cohort

Workplace (occupation)	N (%)	Males, N (%)*	Age at inclusion, years*	Years in service at inclusion*	Daily smokers, N (%)*	Subjects with diagnoses, N (%)*	Mean annual Ni air conc, mg/m^3^
Overall	1424 (100)	1247 (88)	39 (31–47)	14 (8–22)	838 (59)	1215 (85)	-
Smelter	222 (16)	222 (100)	40 (32–47)	16 (11–23)	148 (67)	191 (86)	3.181
Metalworker	209 (15)	209 (100)	39 (32–47)	15 (9–21)	148 (71)	183 (88)	0.734
Electrician	151 (11)	151 (100)	39 (28–46)	14 (6–23)	90 (60)	134 (89)	0.646
Crane operator	128 (9)	51 (40)	40 (33–48)	18 (8–24)	57 (45)	108 (84)	2.375
Burner	120 (8)	120 (100)	37 (31–43)	13 (10–20)	65 (54)	90 (75)	4.202
Welder	70 (5)	70 (100)	33 (27–39)	10 (5–17)	51 (73)	54 (77)	0.568
Metal cleaner	43 (3)	43 (100)	46 (36–50)	20 (11–25)	27 (63)	38 (88)	1.050
Foreman	43 (4)	43 (100)	37 (31–44)	11 (7–16)	26 (60)	36 (84)	1.600
Rigger	38 (3)	38 (100)	45 (33–47)	17 (10–23)	22 (58)	32 (84)	0.950
Crusher operator	37 (2)	28 (76)	46 (36–52)	15 (10–23)	13 (35)	35 (95)	6.760
Batcher	36 (2)	15 (42)	42 (31–49)	12 (7–18)	17 (47)	29 (81)	1.311
Gas purification operator	35 (2)	34 (97)	30 (22–39)	7 (2–14)	19 (54)	23 (66)	1.422
Converter operator	31 (2)	31 (100)	44 (26–49)	17 (3–26)	19 (61)	26 (84)	3.864
Flotation operator	19 (1)	5 (26)	45 (31–49)	14 (9–26)	8 (42)	17 (89)	0.198
Mill operator	19 (1)	19 (100)	42 (26–49)	19 (5–26)	11 (58)	17 (89)	2.856
Others	223 (16)	168 (75)	40 (33–49)	13 (8–21)	117 (52)	202 (91)	-

Note: * - significant differences among occupational groups using Kruskall—Wallis test (for age and years and service) or χ2 test for sex, smoking and subjects with diagnoses

Table 3 shows that we found significant differences in sex, age, years in service, number of smokers and even number of healthy subjects in between-group comparisons using analysis of variance. Gas purification operators and welders represented the youngest staff of the company at the annual screening in 2007, whereas the median age of the oldest occupational groups, including crusher operators and metal cleaners, riggers and flotation operators, was 46 years. Daily cigarette smoking prevalence ranged from 35% in crusher operators, also being the oldest group with the least number of healthy subjects, to 73% in welders, also being the younger occupational group. The median pack-years in 838 daily smokers was 10 (IQR 6–17). Furthermore, the panel of screening doctors verified any chronic condition in 85% workers at the annual screening of 2007, and the number of diagnoses could range from one to eleven in a given worker. The most prevalent chronic condition in the studied cohort of nickel pyrometallurgical production workers were myopia (391 cases), osteochondrosis (242 cases), arterial hypertension (240 cases), chronic bronchitis (191 cases), low back pain (145 cases), nasal septum deviation (142 cases), obesity (134 cases), varicose veins (116 cases), peptic ulcer disease (97 cases) and cervicalgia (80 cases). Table 4 presents the number of cases for each disease class along with the relative measures of disease prevalence at the annual screening.

**Table 4:** Prevalent cases of selected conditions and diseases at the annual screening stratified into classes

Disease class	Cases (%)	Cases per 100 workers
Diseases of the musculoskeletal system and connective tissue	805 (27.8)	56.5
Diseases of the eye and adnexa	646 (22.3)	45.4
Diseases of the respiratory system	460 (15.9)	32.3
Diseases of the circulatory system	382 (13.2)	26.8
Diseases of the digestive system	285 (9.8)	20.0
Endocrine, nutritional and metabolic diseases	198 (6.8)	13.9
Certain infectious and parasitic diseases	155 (5.4)	10.9
Diseases of the genitourinary system	134 (4.6)	9.4
Diseases of the skin and subcutaneous tissue	112 (3.9)	7.9
Diseases of the ear and mastoid process	96 (3.3)	6.7
Neoplasms	84 (2.9)	5.9
Injury, poisoning and certain other consequences of external causes	60 (2.1)	4.2
Diseases of the nervous system	16 (0.6)	1.1
Diseases of the blood and blood-forming organs and certain disorders involving the immune mechanism	13 (0.4)	0.9
Symptoms, signs and abnormal clinical and laboratory findings, not elsewhere classified	9 (0.3)	0.6

Note: data are presented as absolute cases/per cent to all occupational disease claims (N/2895*100) and /cases per 100 workers (N/1424*100)

### Occupational data

Given that the OEL for nickel was 0.05 mg/m^3^, workers in almost all included workplaces were overexposed to insoluble nickel in the air (Table 3). The mean concentrations from a number of samples completed four times a year ranged from 0.198 mg/m^3^ in flotation operators, making this occupational group least exposed, to 6.760 mg/m^3^ in crusher operators. In addition to insoluble nickel in the air, workers in almost all positions were exposed to noise and heat, but the detailed exposure assessment data cannot be provided and analyzed hereinafter.

### Follow-up and occupational diseases

We then followed workers till 2021 with the overall 18,843 person-years of observation. During that time, occupational disease (compensation) claims were confirmed in 129 workers (9% of the initial cohort, N = 108 men (84%)). The median age when an occupational disease was confirmed in these 129 workers was 55 (IQR 50–59) years, with 28.3 ± 6.8 years in service. We found a gradual decrease in the number of workers with confirmed claims during fourteen years of observation (β coefficient − 0.91, p < 0.05) in a linear regression, although the range of workers with new compensation cases a year was wide enough from two workers in 2018 to twenty-two subjects in 2008. Given that the cohort size was gradually decreasing as time elapsed for a variety of reasons, including retirement, promotion to another employer or compensation claim, we also calculated the fraction of occupational disease (compensation) claims in the overall staff annually. As with absolute number of cases per year, this fraction decreased from 1.6 to 1.2% in 2021.

Among 129 workers pursuing compensation and with eventually confirmed occupational disease, 76 workers had occupational chronic bronchitis (3.81 incident cases per 1000 workers per year), 47 subjects had occupational sensorineural deafness (2.36 incident cases per 1000 workers per year), 38 workers had occupational musculoskeletal disorders, including radiculopathies (1.90 incident cases per 1000 workers per year), 27 employees had occupational COPD (1.35 incident cases per 1000 workers per year), and 20 nickel pyrometallurgical workers had occupational asthma (1.00 incident case per 1000 workers per year). There were fewer cases of other work-related diseases, including cancer (5 cases overall or 0.25 incident cases per 1000 workers per year).

We then tested the association of nickel concentrations in the workplace with occupational disease claims. Because nickel concentrations in the workplace for a heterogenous group 16 (“others”) were not available and could not be analyzed, we excluded this occupational group from all further analyses. The mean annual air nickel concentrations in a given workplace were not associated with the risk of an occupational disease (compensation) claim overall. In addition, we could not identify such association for any specific diagnosis from the list of most prevalent diagnoses of occupational diseases, including chronic bronchitis, COPD, asthma, sensorineural deafness or musculoskeletal disorders. We then, however, tested the cut-off air nickel concentrations using ROC analysis and found that the highest sensitivity (0.77) and specificity (0.39) were attributed to air nickel concentrations equal or exceeding 0.95 mg/m^3^ (AUC 0.56; 95% CI 0.51–0.61, p < 0.05). We, therefore, retested the adjusted model with nickel air concentrations as a binary variable (below vs. equal or more than 0.95 mg/m^3^) and found that air nickel concentration equal of above 0.95 mg/m^3^ was a strong predictor (HR 2.12, 95% CI 1.35–3.32) of any occupational disease claim independent of smoking, baseline chronic bronchitis and baseline sensorineural deafness. In addition, in such adjusted Cox model, daily cigarette smoking (HR 2.63 (95% CI 1.53–4.51)), chronic bronchitis at annual screening (HR 4.16 (95% CI 2.81–6.15)) and sensorineural deafness (HR 2.77 (95% CI 1.68–4.58)) were the strongest predictors of any occupational disease (compensation) claims.

### Predictors of occupational diseases

We further studied predictors of each of five most prevalent occupational disease (compensation) claims, including chronic bronchitis, sensorineural deafness, COPD, musculoskeletal disorders and asthma. In an adjusted Cox regression model, smoking was significantly associated with each of five diagnoses with the least effect in sensorineural deafness and the strongest effect in COPD (Table 5). High nickel exposure was associated with occupational bronchitis and occupational asthma. The most powerful predictor of future diagnosis of work-related chronic bronchitis was bronchitis at the annual screening (HR 7.91 (95% CI 4.65–13.45)). Unlike chronic bronchitis, where occupational groups, already adjusted for exposure to nickel, were not associated with the diagnosis, COPD was significantly more often confirmed as a work-related disease in crane operators and batchers independent of smoking. Furthermore, work-related asthma was more often confirmed and received compensation in riggers (HR 4.61 (95% CI 1.29–16.47)) and with even greater effect in flotation operators (HR 30.55 (95% CI 1.88-5.00)), where exposure to nickel was not as high as in many other workplaces (Table 5). As expected, incident occupational claims due to sensorineural deafness were more likely in those who had deafness at the annual screening, and smokers. Moreover, independent of smoking, musculoskeletal disorders of vertebral origin claimed as occupational were more likely to develop in crane operators (HR 5.44 (95% CI 2.23–12.65)) and batchers (HR 4.12 (95% CI 1.22–13.98)). Finally, no associations with the exposure of interest were identified for occupational claims for non-vertebral musculoskeletal diseases (data not shown).

**Table 5:** Hazard ratios of selected predictors of occupational disease (compensation) claims in adjusted Cox regression models for specific diagnoses, which yielded significant associations

	Occupational claim for bronchitis^#^	Occupational claim for COPD^@^	Occupational claim for asthma	Occupational claim for sensorineural deafness	Occupational claim for musculoskeletal disorders of vertebral origin
Smoking	2.56 (1.17–5.57)	6.69 (1.46–30.64)	4.14 (1.17–14.58)	2.84 (1.30–6.19)	3.11 (1.14–8.45)
High nickel exposure	1.92 (1.10–3.35)		7.50 (1.01–57.74)		
Chronic bronchitis	7.91 (4.65–13.45)				
Sensorineural deafness	3.46 (1.93–6.22)			4.12 (3.76–5.86)	
Occupational group 4 (crane operators)		3.35 (1.19–9.49)			5.44 (2.23–12.65)
Occupational group 9 (riggers)			4.61 (1.29–16.47)		
Occupational group 11 (batchers)		5.01 (1.07–23.47)			4.12 (1.22–13.98)
Occupational group 14 (flotation operators)			30.55 (1.88–500)		

*Note: only significant predictors are shown. All hazard ratios with their corresponding 95% confidence intervals are from adjusted Cox regression models. # - model adjusted for all shown predictors; @ - model adjusted for all shown predictors, nickel and baseline COPD. COPD – chronic obstructive pulmonary disease*

When occupational group 4 (crane operators) was combined with smoking, HR of COPD claims was 4.78 (95% CI 1.77–12.90), group 11 (batchers) with smoking – 7.61 (95% CI 1.77–32.71). When group 9 (riggers) was combined with smoking and high nickel exposure, HR of claim of occupational asthma was 7.34 (95% CI 1.67–32.30).

## Discussion

This is the first presentation of the follow-up of a fixed cohort of nickel pyrometallurgical processing workers, showing that occupational diseases diagnosed and confirmed as work-related with the elapsing years of employment were chronic bronchitis, sensorineural deafness, musculoskeletal disorders, COPD and asthma. Respiratory diagnoses led the occupational list, which was indicative of the prevailing inhalational route of occupational risk factors in this production. Smoking was independently associated with all diagnoses, with a greater effect in COPD, whereas high exposure to nickel could independently predict claims for occupational chronic bronchitis and asthma. Associations with pre-existing chronic conditions identified at the annual screening, such as chronic bronchitis and sensorineural deafness, were also confirmed. Selected occupations had a higher chance of future occupational claims, independent of smoking, pre-existing disease and nickel exposure.

The leading occupational exposure in this production was nickel. Despite wide use of metallic nickel in a range of industries and production, occupational health of nickel workers still remains poorly described. Nickel exhibits carcinogenic, toxic and allergic properties, when its toxic mechanism is mediated through cell damage in a number of pathways [12]; therefore, this chemical hazard may affect human health in many ways, also given that exposure limits are likely exceeded in its production sites. Exposure assessment studies, including those not in nickel production, where nickel is somehow used [13, 14], are also quite sporadic and very often outdated [15], but the production technology has likely improved in the last decades and the workers’ exposure may have become less aggressive, but we found no high-quality exposure assessment presentations in the published literature in the last ten years. Unlike electrolytic nickel production, where very few papers portray the picture of the overall exposure [15, 16], pyrometallurgical nickel production is the industry where exposure assessment picture has never been properly demonstrated.

In addition, OEL differ between countries, making compliance testing challenging [17]. In the latter study, as an example, the OEL was 10 times greater for insoluble nickel compounds and 8 times greater for soluble compounds compared to the Russian OELs. In many tests from those production sites, OELs were exceeded. Taken together, this uncovers a large gap in our understanding how much overexposure is present in the nickel production at present and what exactly should be done to reduce such overexposure. High-quality exposure assessment studies are indeed of great need and will guide prevention measure in the future. Furthermore, more effort is needed to refine OELs in the context of how feasible it could be to stay below these limits when economic costs and health consequences are taken together. Exposure data available to us in this presentation demonstrated that compliance with the existing OELs is almost never possible with the technology as it currently stands.

When we consider risk factors of compensation claims in our analysis, cigarette smoking was confirmed as the strongest predictor of occupational claims in all occupational groups of our cohort, consistent with the effect in other occupational groups, including nickel electrolysis [10], gold mining [18, 19], and most other occupations with male predominance and high smoking prevalence. Smoking ban in the workplace may be beneficial not only for smokers, but also for their non-smoking counterparts [19], likely due to discontinuation of exposure to environmental tobacco smoke in the workplace and in living premises in camps. Given that the effect of smoking was the strongest for respiratory disease when the latter were the leading work-related diagnoses in the compensation profile, stricter smoking restriction and ban policy must be prioritized in the workplaces with high exposure to occupational risk factors, such as nickel production. Although we found no published studies on the effect of smoking ban in the nickel production industry, we hypothesized that such interventions would be as beneficial as similar interventions in other industries. Furthermore, employers may ponder to offer discussion leading to subsequent action on the part of an employee to cease smoking, as this is directly associated with costs due to disease in the hazardous workplace and has effect [20–22]. Given that the burden of occupational respiratory disease in the Russian Federation is high [23], targeted interventions for respiratory disease are of great importance.

One of directions to reduce the burden of occupational disease in the nickel industry could be wider use of personal protective equipment (PPE). We could not find any report of the effectiveness of respiratory PPE in the nickel industry, but a few reports from welders showed some effect of PPE on the urine nickel concentration as a marker of exposure [24]. This may be a pivotal health protection measure in nickel production, because inhalation of nickel in large amounts may be fatal, as in a case report of a welder, whose total consumed nickel dose was about 1 gr just within a few hours of welding [25]. Nickel dust fine fractions have been documented to penetrate in the worker’s respiratory tract [26], and assuming that PPE are effective from other industries and field testing is indeed not sufficient as an evidence of worker’s protection, we call for high-quality studies of the effectiveness of PPE in this specific industry, which can provide rationale on whether workers can truly benefit from their use. Respiratory disease predominance in the overall structure of occupational claims in our study only confirms the notion that the respiratory tract is the one most affected and PPE lacks efficiency. Of note, many studies of this intervention effect in other industries rely on a self-report, allowing for some exposure misclassification. We do, however, anticipate studies where particulate matter fractions and concentrations would be measured inside and outside the mask.

In addition to smoking cessation and wider use of PPE, our findings should stipulate further directions of prevention, including technical, organizational and even procedural activities. Firstly, transition to production with lower labor severity should also be set as a health improvement strategy in the long run, because musculoskeletal disorders consistently lead the general morbidity and occupy the second place in the occupational morbidity. Engineering measures like manual labor replacement with automatized processes will help target high musculoskeletal disease incidence. Top three occupations with such high manual labor intensity who need the intervention are smelters, converter operators and burners.

Secondly, hearing protection from excess occupational noise must be set as a priority for immediate technological intervention. There do not exist obstacles to ensure hearing protection of workers which cannot be overcome. Safety regulations in hearing protection can be with the least expenses reinforced and will guarantee significant reduction in exposure. Finally, our findings also support the comprehensive nickel monitoring program, when nickel in blood and urine would be set as a mandatory test of the annual medical screening in all those employed for nickel production. A prospective continuous observation of nickel absorption and excretion in each worker will allow for direct estimation of personal exposure and then find the association with health outcomes.

We could prospectively observe almost all enlisted staff of the production site (93%) and could embrace all occupations in a long follow-up of fourteen years, which was a strength of our analysis. Sex differences in occupational claims were hard to confirm in our presentation due to very small number of employed females, which we considered a limitation. Furthermore, we were limited with poor exposure assessment data, only available from the company internal measurements, but not from the published scientific literature, which we also considered a limitation. These data were hard to reproduce, whereas sampling and laboratory testing methods may have been outdated. Consistent and reproducible exposure assessment data, demonstrating the full magnitude of exposure could have significantly strengthened our analysis. Further research priorities in nickel production should include high-quality and robust exposure monitoring for a more accurate quantification of health effects.

Some misclassification due to poor exposure assessment data was not the only exposure classification bias in our analysis. Baseline diagnoses verification at the annual screening was not complete and often required further tests, but was not accomplished. Such examples included chronic bronchitis, which in many cases back then needed confirmation with further clinical assessment, spirometry follow-up, but instead relied more on the symptoms and self-reported presentation at the annual screening. We also consider this a limitation of our study. Finally, we could not track relocation of workers from one workplace to another during fourteen years of observation, which could have altered exposure pattern; however, such promotion was unlikely in most workplaces.

## Conclusion

In conclusion, this first prospective observation on nickel pyrometallurgical production workers highlighted respiratory disease as the leading cause of occupational disease in need of compensation. Cigarette smoking and high exposure to nickel serve as independent predictors of such claims apart from the occupational groups, and future studies must concentrate on the use of PPE, the effect of smoking cessation and hazard reduction engineering measures. Exposure to both soluble and insoluble nickel salts in this industry should be reduced, and the associated health improvement effects should be documented in future studies.

## Data Availability

The datasets used and/or analysed during the current study are available from the corresponding author on reasonable request.

## List of abbreviations

AUC: Area under curve
CI: Confidence interval
COPD: Chronic obstructive pulmonary disease
HR: Hazard ratio
IQR: Interquartile range
OEL: Occupational exposure limit
PPE: Personal protective equipment
ROC: Receiver operating curve
